# Associations of depression and anxiety with cardiovascular risk among people living with HIV/AIDS in Korea

**DOI:** 10.4178/epih.e2021002

**Published:** 2020-12-24

**Authors:** Kyong Sil Park, Seon Young Hwang, Bo Youl Choi, June Kim, Sang Il Kim, Woo-Joo Kim, Chun Kang

**Affiliations:** 1 School of Nursing, Cheju Halla University, Jeju, Korea; 2 Institute for Health and Society, Hanyang University, Seoul, Korea; 3 School of Nursing, Hanyang University, Seoul, Korea; 4 Department of Preventive Medicine, Hanyang University College of Medicine, Seoul, Korea; 5 Department of Internal Medicine, Yonsei University College of Medicine, Seoul, Korea; 6 Division of Infectious Disease, Seoul St. Mary’s Hospital, College of Medicine, The Catholic University of Korea, Seoul, Korea; 7 Department of Internal Medicine, Korea University College of Medicine, Seoul, Korea; 8 Division of AIDS, Center for Immunology and Pathology, Korea National Institute of Health, Cheongju, Korea

**Keywords:** Acquired immunodeficiency syndrome, Anxiety, Cardiovascular disease, Depressive disorder, Human immunodeficiency virus

## Abstract

**OBJECTIVES:**

As HIV/AIDS is becoming a chronic disease, the risk of developing cardiovascular disease (CVD) among people living with HIV/AIDS is rising. Anxiety and depression, which are common among people living with HIV/AIDS, have been linked with CVD. This study investigated the risk of CVD in people living with HIV/AIDS and explored the effects of depression and anxiety on CVD risk.

**METHODS:**

Data were collected for 457 people enrolled in the Korea Cohort HIV/AIDS study after 2010. Framingham risk scores were calculated to quantify the 10-year risk of developing CVD. Depression and anxiety variables were re-coded as a single combined variable. Multivariable logistic regression analysis was performed, adjusting for age, body mass index, low-density lipoprotein (LDL) cholesterol, triglycerides (TG), duration of human immunodeficiency virus (HIV) positivity after entry into the cohort, and depression/anxiety.

**RESULTS:**

Participants with both depression and anxiety were 2.28 times more likely than those with neither depression nor anxiety to have moderate/high-risk CVD risk. The 10-year risk of developing CVD was affected by LDL cholesterol, TG, age, and duration of HIV infection. LDL cholesterol and TG levels change according to the duration of HIV infection, and metabolic disorders affect the risk of CVD. Thus, a longer duration of HIV infection is associated with a higher risk of developing CVD.

**CONCLUSIONS:**

Screenings for depression and anxiety need to be provided regularly to assess the severity of those symptoms. To help decrease their risk of developing CVD, people living with HIV/AIDS should be offered behavioral modification interventions aimed at developing healthy lifestyle habits.

## INTRODUCTION

Globally, the number of people living with HIV/AIDS has gradually been increasing, while both new infections and deaths due to HIV/AIDS have been decreasing [1].

In Korea, 1,222 new human immunodeficiency virus (HIV) cases were reported in 2019 (1,111 men and 111 women, with a 10:1 male/female ratio). The highest proportion of new cases was found among those in their 20s (35.8%), followed by those in their 30s (27.9%) and those in their 40s (16.5%); altogether, people in their 20s, 30s, and 40s accounted for 80.2% of those living with HIV [2]. Although the number of people living with HIV/AIDS in Korea is not yet a matter of grave concern compared with that in Western countries, this issue still warrants attention because the number of people infected with HIV continues to increase [3].

As the introduction of highly active antiretroviral therapy (HAART) has significantly reduced HIV/AIDS-related mortality rates, people living with HIV/AIDS have become more exposed to the risk of chronic conditions such as cardiovascular disease (CVD) through the aging process [1]. Even with HAART, immune activation persists in people living with HIV/AIDS and may contribute to accelerated atherosclerosis of coronary lesions [4].

According to a systematic literature review, more persistent treatment of HIV increased the relative risk of CVD; specifically, the relative risk among people receiving HAART was approximately 3 times greater than that among people who were not receiving HAART and the relative risk of CVD among HIV-positive people was approximately 61% greater than that among HIV-negative people [5]. Accordingly, comprehensive strategies including early screening and management of risk factors in HIV-positive people are important for the prevention of cardiovascular events [4,6].

A cohort study demonstrated that reducing the cardiovascular risk factor burden may result in substantial reduction in CVD risk among people living with HIV/AIDS [7]. The incidence of CVD is closely related to lifestyle habits such as smoking, insufficient exercise, unhealthy eating habits, stress, and Korean drinking culture for social activities. These lifestyle habits are modifiable risk factors that are common behaviors in middle-aged men and are important in terms of managing CVD [8]. Since people living with HIV/AIDS are gradually aging [9], greater attention should be paid to efforts to reduce the risk factors of CVD among young and middle-aged people living with HIV/AIDS.

Koreans have little knowledge of HIV/AIDS and have negative views of HIV because of the relative lack of social attention paid to HIV compared with Western countries. According to a survey of Koreans on awareness of HIV/AIDS conducted by the Korea Centers for Disease Control and Prevention (KCDC), the idea of acquired immune deficiency syndrome (AIDS) evoked fear, gender, filth/immorality, and disease among the respondents, suggesting that the general public has a negative view of AIDS [9]. People living with HIV/AIDS face frustration and shock due to the severity of the disease. In addition, disruptions in relationships with their families, friends, neighbors, and coworkers can cause not only a loss of emotional stability and self-esteem, but also depression and anxiety [10,11]. A prior study found that people living with HIV/AIDS experienced shock due to HIV infection, desired to hide their HIV infection, feared having other people find out about their HIV infection [12]. Given these difficulties, it is not surprising that many people living with HIV or AIDS may develop depression or anxiety. Another study found that people living with HIV had significantly higher depression/anxiety scores than those who did not [13].

Depression and anxiety are known socio-psychological risk factors for CVD [14], and depression and anxiety symptoms among people with coronary artery disease was found to increase risk for ischemic attack and mortality [15]. In a large cohort study [16], HIV-infected adults with major depressive disorder had a 30% higher risk for myocardial infarction than those without major depressive disorder. As the length of survival in HIV-infected people receiving HAART is increasing, proper attention should be given to their other health issues, as well as to preventing HIV from progressing to AIDS, and socio-psychological factors that can improve their quality of life. Since anxiety and depression are common in people living with HIV/AIDS, and given the association of anxiety and depression with the development of CVD in other chronic illness populations, it is important to study the potential links of anxiety and depression with CVD risk in people living with HIV/AIDS.

This study aimed to determine the risk of developing CVD, and to elucidate the impact of depression and anxiety on the risk of developing CVD, among people living with HIV/AIDS by using baseline data from the Korea Cohort HIV/AIDS (KoCosHIV) study.

## MATERIALS AND METHODS

### Research design

This study adopted a cross-sectional method to determine the impacts of depression and anxiety among people living with HIV/AIDS on the risk of developing CVD by using baseline data from the KoCosHIV study.

### Study participants and data collection

The KoCosHIV study aims to extend HIV-infected patients’ survival time and to improve their quality of life by examining the period from HIV infection to death and identifying risk factors associated with HIV since December 1, 2006 [17]. The inclusion criteria were individuals enrolled in the KoCosHIV study from January 1, 2010 to October 22, 2014, when tests for depression and anxiety were administered (n=660). The exclusion criteria were as follows: (1) individuals who did not answer questions about depression and anxiety (n=60), (2) individuals who did not have available information about gender, age, smoking, diabetes mellitus (DM), total cholesterol, and high-density lipoprotein (HDL) cholesterol, which are needed to calculate the Framingham risk score (FRS) (n=143). In total, 457 study participants were included.

The data for participants were collected via case report forms and blood samples. Information on socio-demographic characteristics, lifestyle factors, anxiety, and depression were collected through a self-report questionnaire. The route of transmission was collected by comparing the self-report questionnaire with the data from the KCDC. Data on DM, hypertension, duration of HIV positivity after entry into the cohort, and HAART at enrollment were collected from hospital records. Information on blood test results and body mass index (BMI) was collected by blood collection and measurements at a hospital. BMI was calculated using height and body weight (kg/m^2^). The collected data were stored en bloc at the KCDC, which builds high quality data through data cleaning. In order to increase the external validity, the research nurses who collected the information were trained by the principal investigator and provided with guidelines.

### Measurements

#### Depression

This study utilized data from the KoCosHIV study to determine levels of depressive symptoms using the Korean version of the Beck Depression Inventory (K-BDI) [18]. It consists of 21 items assessing cognitive, emotional, motivational, and physical symptoms of depression and employs a 4-point Likert scale. The scores range from 0 to 63, with higher scores indicating greater depression. The reliability of the instrument in this study was Cronbach’s α=0.92. Total scores from 0 to 11 are considered normal, those from 12 to 19 indicate mild depression, those from 20 to 35 indicate moderate depression, and those over 36 indicate severe depression. A cut-off score of ≥ 20 was used to identify clinical depression [19].

#### Anxiety

To measure levels of anxiety, we used the Korean version of the State Trait Anxiety Inventory (STAI) [20]. It consists of 20 items assessing levels of anxiety and employs a 4-point Likert scale. The scores range from 20 to 80, with higher scores indicating greater anxiety. The reliability of the instrument in this study was Cronbach’s α=0.89. Total scores from 20 to 51 are considered normal, those from 52 to 56 indicate mild anxiety, those from 57 to 61 indicate moderate anxiety, and those over 62 indicate severe anxiety. A cut-off score of ≥ 57 was used to identify clinical anxiety [20].

#### The 10-year risk of developing cardiovascular disease

The FRS was used to estimate the general 10-year risk of developing CVD [21]. The FRS comprises age, gender, smoking, blood pressure, total cholesterol, HDL cholesterol, and DM based on the sum of the individual risk factor scores. A higher FRS indicates a higher 10-year risk of developing CVD [21,22]. Participants were divided into 3 groups according to their risk of developing CVD: low-risk (< 10%), moderate-risk (10-20%), and high-risk (> 20%) [22].

### Statistical analysis

The collected data were analyzed using SPSS version 21.0 (IBM Corp., Armonk, NY, USA). The characteristics of the participants were analyzed using descriptive statistics, including frequency, percentage, mean, and standard deviation. The main variables of the FRS according to the participants’ 10-year risk of developing CVD were analyzed using descriptive statistics, such as frequency and percentage. The relationships of depression/anxiety, sociodemographic characteristics, and clinical characteristics of the participants with the 10-year risk of developing CVD was analyzed using the independent-samples t-test and the chi-square test. Multivariable logistic regression analysis was conducted for the presence/absence of risk for CVD, adjusting for age, BMI, low-density lipoprotein (LDL) cholesterol, TG, duration of HIV positivity after entry into the cohort, and depression/anxiety. The depression and anxiety variables were re-coded as a single combined variable: neither depression nor anxiety=1, either depression or anxiety=2, and depression and anxiety=3.

### Ethics statement

This study received research approval from the Institutional Review Board (IRB) of Hanyang University and protected participants’ privacy (HYI-15-030-2). The operations committee in charge of the KoCosHIV study evaluated and approved the research plan with the exclusion of participants’ personal information. After each participating hospital received informed consent forms from participants and received approval from its IRB, the research was started.

## RESULTS

### Participants’ characteristics and risk of developing cardiovascular disease

In this study, 94.7% of the participants were men, with a mean age of 40.7± 12.3 years, and the majority of the participants (59.7%) were unmarried. Respectively, 49.0% and 51.5% of the participants currently engaged in smoking and drinking. Almost half (46.4%) of the participants had an infection period of less than 1 year, and the majority (94.1%) had become infected with HIV through sexual contact. At the time of the survey, 70.9% of the participants were receiving HAART and 29.1% were not (Supplementary Materials 1 and 2).

The mean FRS was 2.4± 3.7. The participants were divided into low-risk, moderate-risk, and high-risk groups according to their risk of developing CVD. There were 82 (17.9%) participants in the moderate-risk group and 5 (1.1%) in the high-risk. The average risk of developing CVD was 6.0± 5.1% in the participants of this study. The moderate-risk group consisted of 93.9% men and 6.1% women.

### Differences in participants’ characteristics and depression/anxiety between the low-risk and the moderate/high-risk group of developing cardiovascular disease

**Figure 1.**
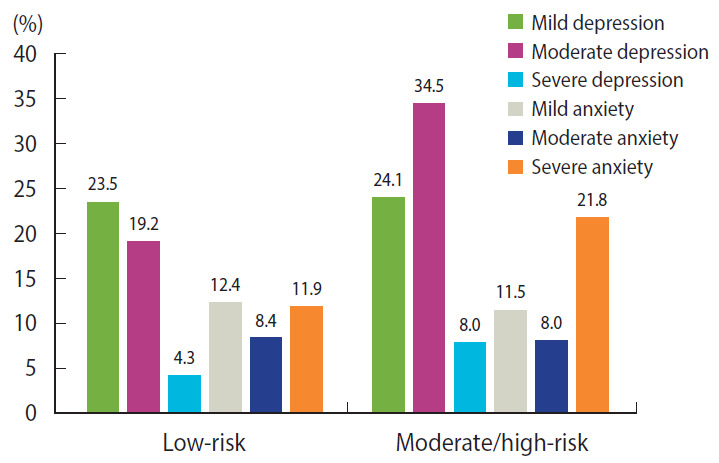
Levels of depression and anxiety according to 10-year cardiovascular risk.Depression was divided into mild depression (12-19 points), moderate depression (20-35 points), and severe depression (≥ 36 points); Anxiety was divided into mild anxiety (52-56 points), moderate anxiety (57-61 points), and severe anxiety (≥62 points); To estimate the 10-year cardiovascular risk, the Framingham risk score (based on age, gender, smoking, blood pressure, total cholesterol, high-density lipoprotein cholesterol, and diabetes mellitus) was calculated, and participants were divided into low-risk (<10%) and moderate/high-risk (≥10%) groups.

Since the high-risk group was small (n=5), the moderate-risk and high-risk groups were combined, and the differences between the low-risk and combined moderate/high-risk group were analyzed. Significant differences were found between the 2 groups in age, BMI, LDL cholesterol, TG levels, the infection period, and the levels of depression and anxiety (p<0.05). A score of 20 or higher and a score of 57 or higher are considered to indicate clinical depression and anxiety, respectively [19,20]. Of the participants with the low-risk group, 23.5% (moderate depression, 19.2%; severe depression, 4.3%) were clinical depression and 20.3% (moderate anxiety, 8.4%; severe anxiety, 11.9%) were clinical anxiety. Of the participants with the moderate/high-risk group, 42.5% (moderate depression, 34.5%; severe depression, 8.0%) were clinical depression and 29.8% (moderate anxiety, 8.0%; severe anxiety, 21.8%) were clinical anxiety (Figure 1 and Table 1).

### Factors affecting the 10-year risk of developing cardiovascular disease

For each 1-year increment in age, participants were 1.17 times more likely to be in the moderate/high-risk group (odds ratio [OR], 1.17; 95% confidence interval [CI], 1.13 to 1.22; p<0.001). For each month of increase in the duration of infection, participants were 1.01 times more likely to be in the moderate/high-risk group (OR, 1.01; 95% CI, 1.00 to 1.02, p=0.005). As the LDL cholesterol level increased by 1 mg/dL participants were 1.03 times more likely to be the moderate/high-risk group (OR, 1.03; 95% CI, 1.02 to 1.04; p<0.001). As the TG level increased by 1 mg/dL, participants were 1.01 times more likely to be in the moderate/high-risk group (OR, 1.01; 95% CI, 1.00 to 1.01; p<0.001). Participants with both depression and anxiety were 2.28 times more likely to be in the moderate/high-risk group than those with neither depression nor anxiety (OR, 2.28; 95% CI, 1.06 to 4.89; p=0.035) (Table 2).

## DISCUSSION

Logistic regression analysis showed that participants with both depression and anxiety were 2.28 times more likely to be in the moderate/high-risk group as those with neither depression nor anxiety. The findings of this study support those of a previous study showing that chronic depressive symptoms increased the risk of developing CVD in HIV-infected women [23] and those of a cohort study showing that people with depression and coronary artery disease had a 31% higher risk for CVD than those without depression [24]. The results of this study are consistent with those of several studies showing that depression and anxiety among people with other diseases predicted CVD [25-27].

The participants in the moderate/high-risk group were more likely to experience clinical depression and anxiety than those in the low-risk group. People are considered clinically depressed when they have K-BDI scores over 20 [19]. In this regard, 23.5% of participants were found to be clinically depressed in low-risk group and 42.5% of participants were found to be clinically depressed in moderate/high-risk group. The level of depression in this study was higher than that reported in several earlier studies conducted abroad addressing the depression levels of people living with HIV/AIDS using the same K-BDI instrument [28-30]. In addition, the percentage of people with clinical depression was higher than 17.9% among HIV-infected patients treated with HAART [29]. People are considered to have clinical anxiety when they have STAI scores over 57 [20]. Using this criterion, 20.3% of participants were found to have clinical anxiety in low-risk group and 29.8%, of participants were found to be clinically anxiety in moderate/high-risk group. In addition, mean of anxiety was 47.14 points in the low-risk group and mean of anxiety was 50.90 points in the moderate/high-risk group. Compared to the anxiety levels of HIV-positive individuals in previous studies using the same STAI instrument, the level found in this study is higher [31]. In a qualitative study in Korea, the lives of people living with HIV/AIDS were found to be characterized by the theme of “living with the yoke of HIV” [12]. A reason why depression and anxiety was more common in this study than in studies of people living with HIV/AIDS conducted abroad could be Korean cultural characteristics, such as the tendency for Koreans to blame themselves for HIV infection and to suppress their feelings. Additionally, they may fear other people finding out about their HIV infection because public awareness of people living with HIV/AIDS is relatively low. Therefore, the pain and fear experienced by people living with HIV/AIDS can cause depression and anxiety. Depression and anxiety may increase the risk of CVD within 10 years. This study supports the need for intervention strategies to manage depression and anxiety among Korean people living with HIV/AIDS.

The group with moderate to high-risk for developing CVD comprised 19.0% of the participants, which is similar to the proportions of 27.7% [32], 22.2% [33], and 16.5% [34] found in previous international studies conducted among men living with HIV. Furthermore, with each 1-increment increase in LDL cholesterol levels, participants became 1.03 times more likely to belong to the moderate/high-risk group. For each 1-increment increase in TG levels, participants were 1.01 times more likely to be in the moderate/high-risk group. These results are in line with previous studies [33,35]. However, BMI did not show a statistically significant relationship. HDL cholesterol levels decrease and TG levels increase in the early stages of HIV infection. However, LDL cholesterol levels decrease in the later stages of HIV infection. Metabolic disorders increase the risk of CVD [36]. Therefore, LDL cholesterol and TG are major risk factors for CVD, and HIV infection causes metabolic disorders, so management of LDL cholesterol and TG levels in people living with HIV/AIDS is essential.

No significant difference between the 2 groups was found in terms of smoking; however, 52.9% of participants in the moderate/highrisk group and 48.1% of those in the low-risk group were current smokers. Therefore, counseling and education about lifestyle modifications are needed, particularly regarding smoking cessation for current male smokers. People living with HIV/AIDS in Korea are predominantly men, with a male-to-female ratio of 10:1; furthermore, more than 80.2% of people living with HIV/AIDS were in their 20s, 30s, and 40s [2]. Thanks to HAART, HIV has become more of a chronic condition, and people living with HIV/AIDS have been more exposed to the risk of CVD due to the aging process [1,4]. Therefore, proactive intervention strategies for modifiable risk factors are needed to prevent and manage CVD efficiently.

In addition, as the infection period extended by 1 month, participants became about 1.01 times more likely to be the moderate/high-risk group. A longer infection period was associated with significantly higher cardiovascular risk in this study. This finding is consistent with previous studies showing that a longer duration of HIV was associated with a higher risk of developing CVD [33]. On the other hand, participants who received HAART did not have a significantly higher risk of developing CVD. This finding is inconsistent with a systematic review on people living with HIV/AIDS showing that those who received HAART were approximately 52% more likely to develop CVD than those who did not [5]. However, because this study analyzed baseline data of the KoCosHIV study using a cross-sectional design, data changes over time could not be examined. Therefore, further research should combine follow-up data with baseline KoCosHIV data to determine how to apply individualized interventions based on the infection period and drug use period by identifying how the risk of developing CVD changes over time.

This study is the first attempt to confirm the relationship between depression/anxiety and the risk of developing CVD using representative data for HIV-infected patients on a large scale from 19 hospitals in Korea. Our findings suggest that HIV/AIDS patients should be regularly screened for depression and anxiety, including assessments of the severity of these symptoms, and those with depression or anxiety should receive counseling to reduce those symptoms. In addition, healthcare providers need to provide motivational education for behavioral modifications to promote good lifestyle habits to decrease patients’ risk of developing CVD, especially among those with depression. Further extensions of this study should focus on more diverse variables affecting the risk of developing CVD, such as changes in depression and anxiety over time, whether HIV-infected patients receive HAART, and drug combinations.

This study has some limitations. First, in this study, causal relationships between the variables cannot be determined because the analysis included baseline data from the KoCosHIV study. Second, this study collected depression and anxiety information using a self-reported questionnaire without proper clinical measurements. Third, this study did not exclude participants with depression and anxiety because it was not possible to investigate their past history of mental disorders. Fourth, this study analyzed only participants enrolled in the KoCosHIV study. Therefore, care should be taken when generalizing the study results to all people living with HIV/AIDS.

## Figures and Tables

**Table 1.** Difference in participants’ characteristics and depression/anxiety in accordance between 10-year cardiovascular risk groups (n=457)

Variables	Category	Low-risk^1^ (<10%, n=370)	Moderate/high-risk^1^ (≥10%, n=87)	t or χ^2^	p-value
Gender	Men	352 (95.1)	81 (93.1)	1.45	0.428
	Women	18 (4.9)	6 (6.9)		
Age (yr)		37.7±11.2	53.2±8.3	-12.11	<0.001
Smoking	Current smoker	178 (48.1)	46 (52.9)	5.67	0.059
	Ex-smoker	66 (17.8)	22 (25.3)		
	Non-smoker	126 (34.1)	19 (21.8)		
Alcohol	Current drinker	195 (53.3)	39 (44.8)	3.79	0.151
	Ex-smoker	68 (18.6)	24 (27.6)		
	Non-drinker	103 (28.1)	24 (27.6)		
BMI (n=437, kg/m^2^)	Normal (<23.0)	229 (65.1)	43 (50.6)	6.99	0.030
	Overweight (23.0-24.9)	68 (19.3)	20 (23.5)		
	Obese (≥25.0)	55 (15.6)	22 (25.9)		
LDL cholesterol (n=440, mg/dL)	<100	223 (62.3)	30 (36.6)	40.36	<0.001
	100-129	106 (29.6)	25 (30.5)		
	130-159	24 (6.7)	21 (25.6)		
	≥160	5 (1.4)	6 (7.3)		
TG (n=454, mg/dL)	<150	211 (57.3)	25 (29.1)	22.32	<0.001
	≥150	157 (42.7)	61 (70.9)		
HAART at enrollment	Yes	258 (69.7)	66 (75.9)	1.28	0.257
	No	112 (30.3)	21 (24.1)		
Duration of HIV positivity after entry to the cohort (yr)	<1	177 (47.8)	35 (40.2)	10.36	0.035
	1-3	80 (21.6)	17 (19.5)		
	3-5	47 (12.7)	9 (10.3)		
	5-7	28 (7.6)	6 (6.9)		
	>7	38 (10.3)	20 (23.0)		
Depression		13.30±10.10	18.40±12.90	-4.01	<0.001
Anxiety		47.14±11.80	50.90±12.30	-2.56	0.012

BMI, body mass index; LDL, low density lipoprotein; TG, triglycerides; HAART, highly active antiretroviral therapy; HIV, human immu nodeficiency virus.

1 Corresponding to the 10-year risk of cardiovascular disease.

**Table 2.** Factors affecting the 10-year risk of developing CVD (n=457)

Variables	n	mean±SD	aOR (95% CI)^1^	p-value
Age (yr)	457	40.7±12.3	1.17 (1.13, 1.22)	<0.001
BMI (kg/m^2^)	437	22.3±3.1	1.12 (0.99, 1.24)	0.070
LDL cholesterol (mg/dL)	440	96.0±30.5	1.03 (1.02, 1.04)	<0.001
TG (mg/dL)	454	180.8±124.9	1.01 (1.00, 1.01)	<0.001
Duration of HIV positivity after entry to cohort (mo)	457	32.14±40.49	1.01 (1.00, 1.02)	0.005
Depression/anxiety				
Neither depression nor anxiety	306	-	1.00 (reference)	
Depression or anxiety	77	-	2.05 (0.95, 4.44)	0.069
Both depression and anxiety	74	-	2.28 (1.06, 4.89)	0.035

CVD, cardiovascular disease; SD, standard deviation; aOR, adjusted odds ratio; CI, confidence interval; BMI, body mass index; LDL, low-density lipoprotein; TG, triglycerides; HIV, human immunodeficiency virus.

1 Adjusted for age, BMI, LDL cholesterol, TG, duration of HIV positivity after entry into the cohort, and depression/anxiety.

## Supplementary Materials

Supplementary Material 1. Socio-demographic characteristics of subjects

Supplementary Material 2. Clinical characteristics of subjects
