# Supplementary material for: Associations of depression and anxiety with cardiovascular risk among people living with HIV/AIDS in Korea
Source: Epidemiol Health. 2020 Dec 24;43:e2021002. doi: 10.4178/epih.e2021002 (PMC7952836; doi:10.4178/epih.e2021002)
Supplement: Supplementary Material 1. — Socio-demographic characteristics of subjects [file epih-43-e2021002-suppl1.pdf]

Volume: 43, Article ID: e2021002

https://doi.org/10.4178/epih.e2021002

Supplementary Material 1. Socio-demographic characteristics of subjects (N=457)

Variable	Category	n (%)	M±SD (range)
Gender	Men	433 (94.7)	
	Women	24 (5.3)	
Age (yr)	18–29	104 (22.8)	40.7±12.3 (18-73)
	30–39	108 (23.6)	
	40–49	136 (29.8)	
	50–59	76 (16.6)	
	60–73	33 (7.2)	
Marital status	Single	273 (59.7)	
	Married/Living together	118 (25.8)	
	Separated/Divorced/Widowed	66 (14.4)	
Smoking	Current smoker	224 (49.0)	
	Ex-smoker	88 (19.3)	
	Non-smoker	145 (31.7)	
Alcohol	Current drinker	234 (51.2)	
	Ex-drinker	92 (20.1)	
	Non-drinker	127 (27.8)	
Past medical history[1]	Hypertension	49 (10.7)	
	Dyslipidemia	47 (10.3)	
	Diabetes mellitus	27 (5.9)	
	Lipodystrophy	10 (2.2)	
Family CVD history[1] (n=447)	Hypertension	136 (30.4)	
	Diabetes mellitus	109 (24.4)	
	Stroke	35 (7.8)	
	Ischemic heart disease	18 (4.0)	
	Dyslipidemia	6 (1.3)	
	Peripheral vascular disease	1 (0.2)	
BMI (kg/㎡) (n=437)	Normal (<23)	272 (62.0)	22.3±3.1 (14.5–37.8)
	Overweight (23–24.9)	88 (19.3)	
	Obese (≥25)	77 (17.5)	

BMI, body mass index; CVD, cardiovascular disease.

[1]Multiple responses
