# Supplementary material for: Associations of depression and anxiety with cardiovascular risk among people living with HIV/AIDS in Korea
Source: Epidemiol Health. 2020 Dec 24;43:e2021002. doi: 10.4178/epih.e2021002 (PMC7952836; doi:10.4178/epih.e2021002)
Supplement: Supplementary Material 2. — Clinical characteristics of subjects [file epih-43-e2021002-suppl2.pdf]

Volume: 43, Article ID: e2021002

https://doi.org/10.4178/epih.e2021002

Supplementary Material 2. Clinical characteristics of subjects (N=457)

Variable	Category	n (%)	M±SD (range)
Total cholesterol (mg/dL)	<200	359 (78.6)	170.0±39.7 (61.0–300.0)
	200–239	70 (15.3)	
	≥240	28 (6.1)	
HDL-cholesterol (mg/dL)	<40	197 (43.1)	43.0±14.7 (4.0–145.0)
	40–59	220 (48.1)	
	≥60	40 (8.8)	
LDL-cholesterol (mg/dL, n=440)	<100	253 (57.5)	96.0±30.5 (10.0–210.0)
	100–129	131 (29.8)	
	130–159	45 (10.2)	
	≥160	14 (3.1)	
TG (mg/dL, n=454)	<150	236 (52.0)	180.8±124.9 (14.3–1035.0)
	≥150	218 (48.0)	
Duration of HIV positivity after entry to cohort (yr)	<1	212 (46.4)	2.7±3.4 (0–17.6)
	1–3	97 (21.2)	
	3–5	56 (12.3)	
	5–7	34 (7.4)	
	>7	58 (12.7)	
Route of transmission[1]	Sexual contact (n=375)	413 (94.1)	
	Heterosexual contact	188 (50.1)	
	Homosexual contact	157 (41.9)	
	Bisexual contact	30 (8.0)	
	Etc[2]	15 (3.4)	
HAART at enrollment	Yes	324 (70.9)	
	No	133 (29.1)	
CD4+ cell count at enrollment (cells/㎣)	<200	99 (21.7)	390.9±250.3 (0.0–1,646)
	200–349	124 (27.1)	
	350–499	117 (25.6)	
	≥500	117 (25.6)	
Viral load at enrollment (copies/㎖)	≤500	252 (55.1)	101,030±864,165.2 (0.0–17,780,000)
	501–3,000	25 (5.5)	
	3,001–10,000	26 (5.7)	
	10,001–30,000	34 (7.4)	
	>30,000	120 (26.3)	

HDL, high density lipoprotein; LDL, low density lipoprotein; TG, Triglycerides; HAART, highly active antiretroviral therapy; CD, cluster of differenciation

[1]Multiple responses

[2]Etc: piercing, acupuncture, dental treatment, medical appliances
